# Polarized Macrophages Show Diverse Pro-Angiogenic Characteristics Under Normo- and Hyperglycemic Conditions

**DOI:** 10.3390/ijms26104846

**Published:** 2025-05-19

**Authors:** Mahnaz Shariatzadeh, César Payán-Gómez, Julia Kzhyshkowska, Willem A. Dik, Pieter J. M. Leenen

**Affiliations:** 1 Department of Immunology, Erasmus University Medical Center, 3015 GD Rotterdam, The Netherlands; 2 Academic Direction, Universidad Nacional de Colombia, Sede de La Paz, Cesar 202010, Colombia; cepayang@unal.edu.co; 3 Institute of Transfusion Medicine and Immunology, Institute for Innate Immunoscience (MI3), Medical Faculty Mannheim, University of Heidelberg, 68167 Mannheim, Germany; julia.kzhyshkowska@medma.uni-heidelberg.de; 4 German Red Cross Blood Service Baden-Württemberg—Hessen, 89081 Ulm, Germany; 5 Laboratory Medical Immunology, Department of Immunology, Erasmus University Medical Center, 3015 GD Rotterdam, The Netherlands; w.dik@erasmusmc.nl

**Keywords:** macrophage polarization, angiogenesis, gene expression, tumor-associated macrophages, hyperglycemia

## Abstract

Angiogenesis plays a crucial role in solid tumor growth. Ischemia and inflammation induce various angiogenic mediators, and patient metabolic conditions importantly influence this process. Macrophages closely interact with the vascular system and regulate angiogenesis through pro/anti-angiogenic factors. Traditionally, pro-angiogenic activity has been attributed to M2-like macrophages. We question this, as recent evidence suggests that also M1-like macrophages can be pro-angiogenic. Therefore, the aim is to identify the pro/anti-angiogenic gene expression profiles of human polarized macrophages unbiasedly. We also examine the effect of hyperglycemia on angiogenic gene expression, reflecting its role in diabetes and other metabolic conditions. Bioinformatic analysis was performed on the angiogenesis-related gene expression profiles of CD14+ monocyte-derived M1(IFN-γ)- and M2(IL-4)-polarized macrophages. The top differentially expressed genes were selected for validation. Macrophages were generated in vitro and polarized to M1(IFN-γ) and M2(IL-4/IL-6) cells under standard/hyperglycemic conditions. After immunophenotypic confirmation, selected gene expression was quantified using qPCR. IL-4 and IL-6 induce distinct M2-like phenotypes with mixed pro/anti-angiogenic gene expression. Remarkably, IFN-γ stimulation also increases several pro-angiogenic genes. Hyperglycemia affects the angiogenic expression profile in both M1- and M2-like macrophages, although distinctive identities remain intact. The pro-angiogenic phenotype is not limited to M2-polarized macrophages. Both M1- and M2-like macrophages express complex pro/anti-angiogenic gene profiles, which are only mildly influenced by hyperglycemia.

## 1. Introduction

Angiogenesis is the crucial process through which new blood vessels are formed from pre-existing vasculature [1]. Physiological angiogenesis is typically ischemia-driven by HIF-1α activation, stimulating production of angiogenic mediators, in particular, VEGF-A. In contrast, pathological angiogenesis, characterized by the formation of abnormal and dysfunctional blood vessels, is associated with inflammation [1,2], driven by the proliferative and migratory response of endothelial cells to inflammatory cytokines. These are mostly produced locally by activated tissue-resident and infiltrating macrophages. Ischemia and inflammation are both processes characterizing solid tumors [3]; therefore, investigating the triggers stimulating and regulating angiogenesis is highly relevant for the understanding of tumor angiogenesis.

Macrophages exhibit a nuanced interplay with the vascular system and serve as crucial modulators of angiogenesis [4,5,6]. Macrophages can either promote or inhibit angiogenesis depending on their activation state and the microenvironmental signals [7]. The functional diversity of macrophages stems from their plasticity and ability to differentiate into several subsets with varying properties [8,9]. Based on microenvironmental cues, myelomonocytic cells polarize into a flexible spectrum of different polarities, with tissue- and context-specific properties [7]. Traditionally, M1-like macrophages are linked to classical activation and pro-inflammatory responses, while M2-like macrophages are associated with alternative activation, anti-inflammatory responses, and pro-angiogenic activity [10,11,12,13,14,15]. This alignment seems logical at first glance; however, emerging evidence challenges the exclusive association of pro-angiogenic activity with the M2 phenotype, in line with the notion that pathological angiogenesis is driven by inflammation, which is typically linked to M1-like macrophage polarization [2,16]. In accordance, Hagbi-Levi et al. found pro-angiogenic activity particularly associated with (IFN-γ + LPS)-stimulated, i.e., M1-polarized macrophages [17]. Therefore, the insight into the angiogenic profile of polarized macrophages is at least confusing and needs clarification in an unbiased approach.

In the context of diabetes, there is evidence of a low-grade chronic inflammation indicated by the accumulation of monocytes and granulocytes into tissues such as the retina, which has been associated with vascular dysfunction [18,19]. Hyperglycemia, a hallmark of diabetes, has important consequences for angiogenesis and wound healing and induces a mixed phenotype differentiation of macrophages, including both M1- and M2-associated markers [20]. Metabolic conditions support several cancer types where angiogenesis is a pivotal process to provide growing tumor mass with oxygen and nutrition. Angiogenesis is also crucial for cancer cells to enter the blood circulation and to build distant metastases. Tumor-associated macrophages (TAMs) are essential for the angiogenic switch and produce various factors that support tumor angiogenesis [5,21]. At the same time, macrophage activation is controlled by metabolic conditions, and in particular, by hyperglycemia [20,22,23]. However, the extent and the role of metabolic conditions in programming macrophages to support or inhibit angiogenesis is an open question.

In this study, we aim to understand the relationship between human macrophage polarization and their expression of pro- and anti-angiogenic factors in an unbiased manner. To achieve this, we performed a comprehensive bioinformatic analysis of the pro- and anti-angiogenic gene expression profiles of M1- vs. M2-polarized macrophages, based on our previously published dataset [20]. To substantiate our findings, we have differentiated and polarized macrophages into M0-, M1-, and M2-like macrophages under standard (11 mM) or high glucose (25 mM) conditions and conducted an unbiased validation of the differentially expressed genes (DEGs) retrieved from the aforementioned data set.

## 2. Results

### 2.1. Basic Bioinformatic Analysis of Macrophages Polarized Under Normo- and Hyperglycemic Conditions

After data curation from our previously published dataset, we first calculated the numbers of differentially expressed genes (DEGs) between polarized macrophages under normal (5 mM; NG) and hyperglycemic (25 mM; HG) conditions (Table 1). Essentially similar numbers of up- and down-regulated genes were observed in both M1- and M2-polarized macrophages compared to unpolarized cells (M1-NG vs. M0-NG and M2-NG vs. M0-NG). Exposure to high levels of glucose resulted in partial modifications to the transcriptome. Specifically, in M1, 563 genes (1753 minus 1190; 32% of DEG) were uniquely altered in M1-HG vs. M0-NG, and in M2, 233 genes (1421 minus 1188; 26% of DEG) were exclusively differentially expressed in M2-HG vs. M0-NG but not in M2-NG vs. M0-NG.

The biological interpretation of the list of DEGs was performed using the over-representation analysis implemented in DAVID. Table 2A,B shows the top 10 regulated pathways induced by M1 differentiation under NG and HG conditions, respectively. Interestingly, ‘Angiogenesis’ (GO:0001525) was the eighth most prominent pathway. Under HG conditions, similar pathways were regulated as under NG conditions, with a predominance of regulated immune system pathways. Interestingly, compared to M0-NG, the angiogenesis pathway showed a higher number of DEGs (41 vs. 38) and was identified as the second most important (Table 2B). Analysis for M2 in NG and HG conditions compared to M0 showed similar immune system-related pathways most prominently involved (Table 3A,B). The angiogenesis pathway was ranked lower in the M2-modulated pathways but still statistically significant. Taken together, pathway analysis indicated that angiogenesis-related genes are modulated upon both M1 and M2 polarization of macrophages in a quantitatively similar fashion and under both normal or elevated glucose levels.

### 2.2. Identification of the Pro- and Anti-Angiogenic Genes Regulated upon M1 and M2 Macrophage Polarization

Next, we investigated in the same dataset whether pro- and anti-angiogenic genes were differentially regulated upon IFN-γ vs. IL-4 stimulation and whether glucose levels influenced this. To that end, we used the set of pro- and anti-angiogenic genes annotated as such in the Gene Ontology and Ingenuity Pathway Analysis databases. The pro-angiogenic pathway consisted of 301 genes, while the anti-angiogenic pathway had 114 genes (Supplementary Table S1). Table 4 shows that M1 polarization under normoglycemic conditions caused significant up-regulation of 22 pro-angiogenic genes (7%) and down-regulation of 39 (13%) compared to unpolarized cells. M2 polarization, on the other hand, mediated up-regulation of 41 pro-angiogenic genes (14%) and down-regulation of 30 (10%). For anti-angiogenic genes, M1 polarization showed a balanced up- and down-regulation (8 (7%) vs. 9 (8%)), while M2 polarization mediated down-regulation of 21 anti-angiogenic genes (18%), but up-regulation of only 12 genes (11%). Together, this indicates that only minor fractions (7–18%) of pro- and anti-angiogenic genes are significantly up- or down-regulated upon either M1- or M2-polarization. Stimulation with IL-4 caused an overall pro-angiogenic balance, while IFN-γ stimulation showed a mixed response.

Table 5 shows the effects of hyperglycemia upon M1- or M2-polarization. In M1(IFN-γ)-stimulated cells, more pro-angiogenic genes are up- than down-regulated when hyperglycemic conditions are compared to normoglycemia (48 (16%) up vs. 28 (9%) down), while IL-4 stimulation caused more down- than up-regulation (65 (22%) down vs. 50 (17%) up). For anti-angiogenic genes, the hyperglycemia effects were in similar ranges for M1- and M2-polarized cells (13–19%). Thus, hyperglycemia has effects on angiogenic gene expression in both M1- and M2-polarized macrophages, with a net stimulation of angiogenic genes in M1-like cells and a net inhibition in M2-like cells. These data need to be interpreted with great caution, however, since contributions of individual genes likely differ greatly in angiogenesis.

**Figure 1:**
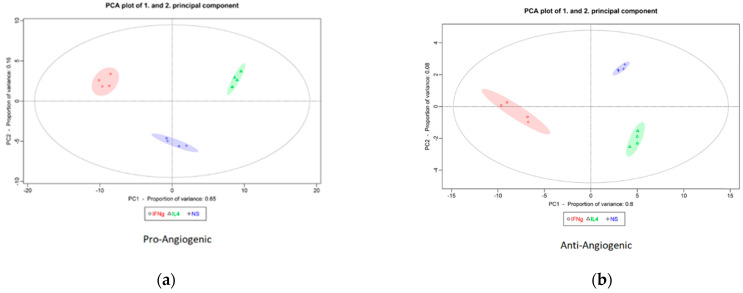
PCA of M0, M1(IFN-γ), and M2(IL-4) macrophages using the expression profiles of pro-angiogenic (**a**) and anti-angiogenic (**b**) genes. Both figures show three clusters: blue—M0 unpolarized cells, red—M1(IFN-γ) cells, and green—M2(IL-4) cells.

To visualize the differences between differently polarized macrophages regarding expression of pro- and anti-angiogenic genes under normoglycemic conditions, we performed PCA analysis (Figure 1a,b, respectively). Concerning pro-angiogenic genes, in PC1 (representing 65% of diversity), M0 cells are intermediate between M1 and M2 cells. With regard to anti-angiogenic genes, however, M0 and M2 cells show a similar PC1 position (80% of diversity), while M1 cells stand apart in this respect. These results indicate that M1 and M2 cells exhibit clear differences in the levels of angiogenesis-related gene expression compared to each other and to M0, as expected.

To enable in vitro validation of these in silico results, we subsequently selected the set of most prominently expressed and regulated angiogenesis-related genes out of those revealed from PCA analysis. Utilizing the PCAGoPromotor package, we ranked these genes based on their individual importance in shaping PC1 and PC2 within each PCA depicted in Figure 1. This ranking considered the significance of these genes in both the positive and negative segments of PC1 and PC2, as detailed in Supplementary Table S3. Subsequently, we selected the top three genes from PC1 and the top two genes from PC2 for both the pro-angiogenic and anti-angiogenic gene lists (Supplementary Table S3). Notably, we included *VEGFA* in the list of pro-angiogenic genes, as it serves as a crucial angiogenesis-stimulating factor that exhibited up-regulation in M1 and M2 cells when compared to M0. In annotation, *VEGFA* is listed as having both pro- and anti-angiogenic activity. Albeit indeed specific VEGF-A isoforms have anti-angiogenic capacity [24], we consider VEGF-A production by macrophages primarily as a pro-angiogenic feature [25].

### 2.3. In Vitro Validation of Angiogenic Gene Expression of M1 and M2 Differentiation Under Standard and Hyperglycemic Conditions

To validate the outcomes of the bioinformatic analysis of angiogenic expression profiles from the published dataset, CD14^+^ monocytes were differentiated under standard (11 mM) or high glucose (25 mM) culture conditions and polarized into M1 (IFN-γ stimulation) or M2 phenotypes (IL-4 or IL-6 stimulation). We used 11 mM as the standard glucose concentrations, as glucose levels in culture medium decline significantly over time due to cellular consumption. Furthermore, M1 macrophages, which are predominantly glycolytic, rapidly deplete glucose, leading to early nutrient limitation. Therefore, 11 mM was chosen to ensure a more stable and comparable metabolic environment over the course of the differentiation and polarization process, while still representing a condition distinct from the used hyperglycemia. Flow cytometry analysis confirmed that M1(IFN-γ)- and M2(IL-4 or IL-6)-polarized macrophages showed the expected phenotype and marker expression, as reported previously by others (Supplementary Figures S1 and S2) [26]. IL-6-stimulated macrophages expressed higher levels of M2-associated markers CD16, CD163, and CD206 compared to IFN-γ- and IL-4-induced macrophages, but variations in responses among donors caused them not to differ significantly from the M0 phenotype (Supplementary Figure S1b). High glucose concentration slightly attenuated the expression levels of these markers in both IL-6-induced and M0 macrophages.

**Figure 2:**
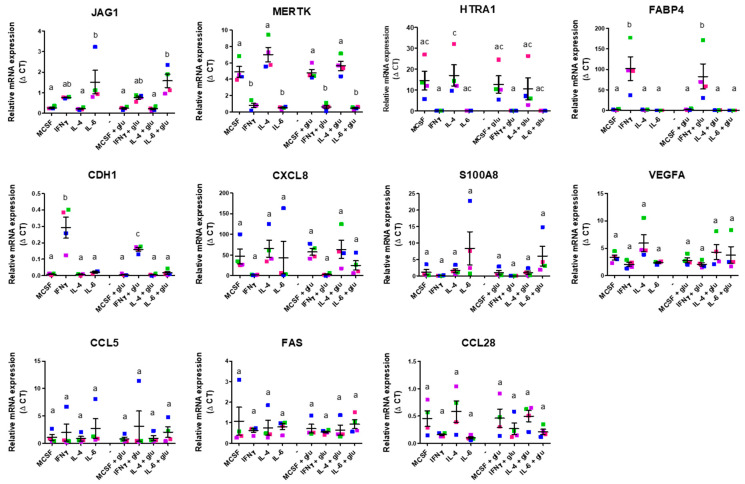
In vitro validation of pro-angiogenic gene expression of M0-, M1-, and M2-polarized macrophages. Expression of pro-angiogenic genes by different macrophage subsets differentiated in the presence and absence of elevated glucose concentration for 4 days. Expression is depicted as relative mRNA expression. Error bars represent means ± standard error of the mean (SEM). Different letters indicate significant differences: groups not sharing a letter differ significantly from each other. For example, if group A is labeled with “a” and group B with “b”, the difference between them is statistically significant; if both share the same letter (e.g., “ab”), the difference is not significant. Significance was calculated using one-way ANOVA and Tukey post-hoc test correction for multiple comparisons (*p* < 0.05). Differently colored symbols indicate results from different monocyte donors.

**Figure 3:**
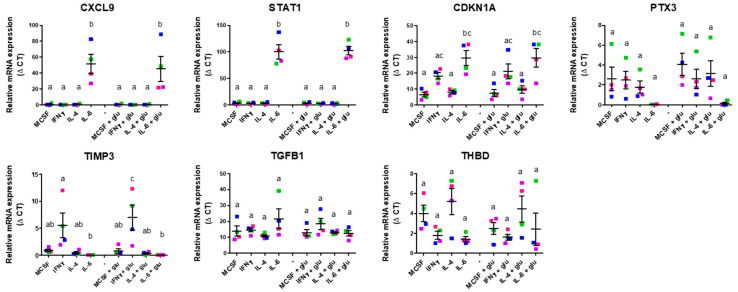
In vitro validation of anti-angiogenic gene expression of M0-, M1-, and M2-polarized macrophages. Expression of anti-angiogenic genes by different macrophage subsets differentiated in the presence and absence of elevated glucose concentrations for 4 days. Expression is depicted as relative mRNA expression. Error bars represent means ± standard error of the mean (SEM). Different letters indicate significant differences. Significance was calculated using one-way ANOVA and Tukey post-hoc test correction for multiple comparisons (*p* < 0.05). Differently colored symbols indicate results from different monocyte donors.

Subsequently, we assessed the expression levels of pro- and anti-angiogenic genes in these distinct macrophage subsets polarized in standard or high-glucose culture conditions using qPCR. These results are shown in Figure 2 and Figure 3, respectively.

Analyzing expressions of pro-angiogenic genes (Figure 2), we observed heterogeneous profiles induced by the various conditions. For instance, *JAG-1* expression was only significantly elevated in IL-6-induced macrophages compared to M-CSF-stimulated controls. *MERTK* and *HTRA1* expression levels were decreased in IFN-γ- and IL-6-induced macrophages compared to the other subsets. In contrast, only IFN-γ stimulation resulted in increased expression of *FABP4* and *CDH1*. Substantial variation among different donors prevented the expression level differences between distinct subsets from reaching significance for *CXCL8* (*IL-8*), *S100A8*, *VEGFA*, *CCL5*, *FAS*, and *CCL28* among the different macrophage subtypes.

Among the anti-angiogenic genes expressed by differentially polarized macrophages, similarly diverse profiles were observed, also with variation among individual samples, thus limiting statistical power. *CXCL9*, *STAT1*, and *CDKN1A* were more highly expressed, and *PTX3* expression was lower in IL-6-stimulated macrophages in comparison with other macrophage subsets, regardless of glucose concentration (Figure 3). Furthermore, *CDKN1A* expression was also stimulated in IFN-γ-differentiated macrophages, albeit to a lesser extent than by IL-6. Notably, IFN-γ-induced macrophages exhibited a relatively higher expression of *TIMP3*. Conversely, no significant discernable changes in the expression levels of *TGFB1* and *THBD* were observed among the different macrophage subsets. Together, these data represent a complex angiogenic profile that incorporates both pro- and anti-angiogenic characteristics across all M0, M1, and M2 macrophage phenotypes.

**Figure 4:**
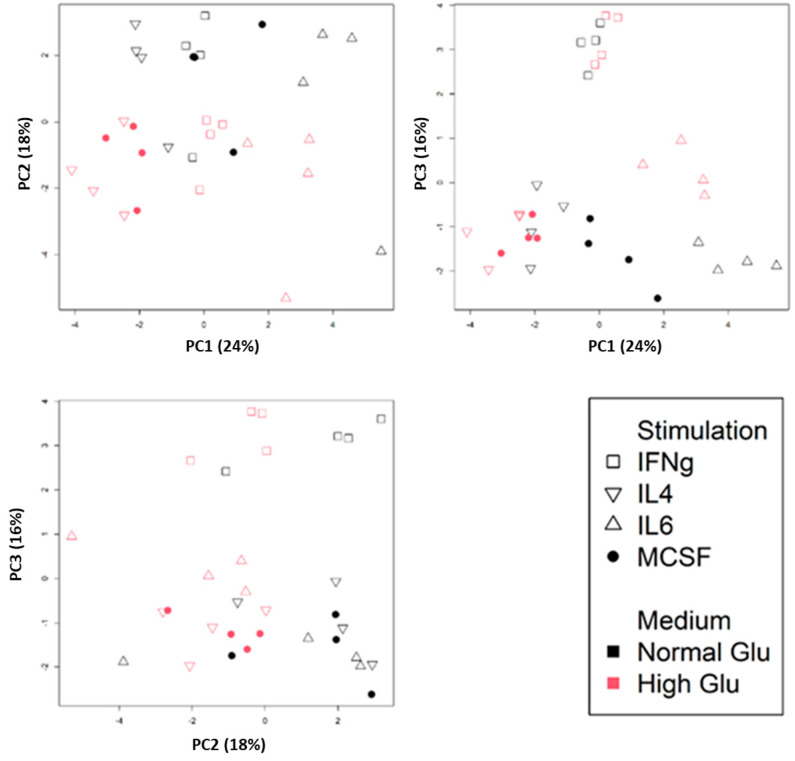
Principal component analysis of polarized macrophages based on immunophenotypes determined by flow cytometry and qPCR data obtained in the validation experiments on expression of angiogenesis-related factors. Three figures show PC1-PC2, PC1-PC3, and PC2-PC3, respectively. PC values of individual samples are shown. Red represents a high glucose condition.

To visualize the integrated results of macrophage immunophenotype and angiogenesis-related gene expression, we conducted PCA on the combined phenotypic and gene expression datasets obtained in the validation experiments (Figure 4). Despite considerable variation, the individual subtypes could be clearly distinguished as clusters based on three distinct components. PC1 primarily discriminated the various polarized subtypes, while PC2 facilitated a separation between macrophages generated under standard and high glucose conditions, with HLA-DR, *CDH1*, and *FAPB4* being the most important contributing factors. Remarkably, IL-6-stimulated macrophages exhibited the greatest dissimilarity in PC1 from the macrophages stimulated by IL-4, although both are considered M2-like cells. M0 and IFN-γ-stimulated M1 macrophages take on an intermediate position in this respect. Furthermore, PC3 distinguished clusters that were specific to macrophages induced by IFN-γ stimulation.

## 3. Discussion

In this study, we aimed to interrogate, in an unbiased manner, the notion that pro-angiogenic activity of macrophages is generally ascribed to M2-polarized macrophages. Furthermore, we examined the impact of hyperglycemia on such macrophage activity, given the prominent angiogenesis-related complications in diabetes. Our findings revealed that both M1(IFN-γ)- and M2(IL-4)-polarized subtypes exhibit varying expression levels of pro- and anti-angiogenic genes, with similar differences between these types in their overall angiogenic profiles compared to unpolarized macrophages. Cells generated under hyperglycemic conditions differed from those generated under standard glycemia, based on phenotypic and angiogenic gene expression patterns, generally showing a diminished polarized response.

The constraints of the M1/M2 paradigm in describing macrophage polarization are increasingly becoming evident, as these entities delineate opposite ends of a spectrum that are not typically observed in vivo [9,27,28]. Recent studies at the single-cell level have investigated how cytokines and pathogen signals impact macrophage phenotypes and challenged the paradigm of distinct subsets based on a limited set of selected ligands in the immune response [7,28]. Instead, they suggest there are common inflammatory pathways that interact to form complex and even mixed phenotypes of macrophages, also depending on the cellular origin. These insights align with the growing recognition that origin, environment, and time collectively shape macrophage identity, as seen in TAMs [29]. By integrating temporal aspects into macrophage classification, researchers can better understand the continuum between metabolic diseases, inflammation, and cancer, further refining the dynamic framework beyond traditional M1/M2 definitions. As a next step, machine learning approaches now define patterns across populations from different origins, thus delineating macrophages in different phases of ‘reactive’ and ‘tolerant’ states [9].

Angiogenesis is regulated by various factors, with ischemia and inflammation playing key roles as drivers. Under hypoxic conditions, such as in tumors, macrophages are often activated in a pro-inflammatory manner yet are pro-angiogenic in nature, thus challenging the generally accepted association of M2 polarization and stimulation of angiogenesis [1,30]. For instance, inflammatory IFN-γ-stimulated macrophages, particularly from patients with age-related macular degeneration, show typical M1-like characteristics and are markedly pro-angiogenic, even more than IL-4-stimulated macrophages [17]. These findings, along with the mixed responses observed in our bioinformatic analysis and the similarly varied outcomes from qPCR validation, highlight a more complex relationship between macrophage polarization and pro-angiogenic activity. Accordingly, we suggest that the general notion linking pro-angiogenic functionality exclusively to M2 polarization is misconceived, and both M1- and M2-polarized macrophages might exert pro-angiogenic characteristics, albeit via different routes, depending on their activation state. Notably, this concept aligns with TAMs, which can display pro-angiogenic activity independent of classical M2 polarization, particularly in the presence of tumor-driven metabolic rewiring [31].

We utilized different stimuli to induce M2-like polarization, IL-4 and IL-6, which showed distinct alterations in immunophenotype and in expression of angiogenesis-related genes compared to M0 macrophages. In particular, IL-6 induced a unique profile as it increased expression of *JAG1* (*Jagged1*), a pro-angiogenic factor and Notch ligand [32]. Simultaneously, IL-6 up-regulated anti-angiogenic factors *STAT1* and *CXCL9*, both in standard and high glucose conditions. Additionally, and in line with previous studies [33,34], IL-6 stimulation increased the expression of anti-angiogenic *CDKN1A*, a key regulator of cell cycle progression. It appears that IL-6-induced macrophages display a phenotype sharing traits with both M0- and M2-like macrophages but exhibit a distinct pro-inflammatory phenotype with mixed angiogenic features. This corresponds with findings on angiogenic TAMs, which can arise from inflammatory TAMs under hypoxic conditions and tumor-induced VEGF-A signaling [35]. In multiple cancers, tumor cores enriched with macrophages exhibit overlapping inflammatory and angiogenic signatures, further supporting the notion that inflammatory stimuli like IL-6 can drive macrophages toward a hybrid pro-inflammatory and pro-angiogenic state. Earlier studies show that IL-6 promotes VEGF expression and angiogenesis in various tissues, including the brain, largely through its action on tissue macrophages [36,37,38]. However, IL-6-driven angiogenesis often results in structurally and functionally abnormal vessels, suggesting their role in pathological angiogenesis [39,40]. The transcriptomic analyses across diverse macrophage polarization states also revealed a strong correlation between IL-6 and VEGF expression, indicating a shared inflammatory-angiogenic axis. Moreover, IL-6 has been shown to drive macrophages toward an unconventional, pro-inflammatory M2-like phenotype, as seen in aging and obesity, where IL-6-rich macrophages contribute to vascular dysfunction and chronic inflammation [41,42,43,44]. Consistent with these findings, our data show that IL-6–stimulated macrophages do not represent a classical M2 phenotype but rather adopt a distinct transcriptional profile. This is also supported by our PCA results, which clearly show that IL-6–stimulated cells cluster separately from IL-4–polarized M2 macrophages, reinforcing the notion that IL-6 induces a unique macrophage phenotype with mixed characteristics.

Our current study revealed a slight inhibitory effect of hyperglycemia on the expression of M2-associated surface markers on macrophages, particularly in IL-6-induced macrophages. This is likely associated with the recently found elevated expression of TLRs and enhanced TLR-mediated inflammatory response in these cells in hyperglycemic conditions [23]. However, this effect did not translate into profound changes in the angiogenic gene expression profile of the macrophages as observed in our qPCR validation experiments. This might be attributed to the short duration of exposure to hyperglycemia in our experimental setup, as compared to the chronic hyperglycemia in in vivo diabetic conditions. The latter may lead to epigenetic modifications in macrophages and altered expression of inflammatory/angiogenic genes [45]. Hyperglycemia, in the absence of additional metabolic factors, drives a mixed M1/M2 differentiation profile characterized by the production of cytokines that can play a critical role in insulin resistance, diabetes-associated inflammation, vascular complications, and tumor progression [20]. It should be emphasized, however, that hyperglycemia is well-established to exert potent pro-inflammatory effects on immune cells, affecting both innate and adaptive immunity [46,47].

Our study has certain limitations that should be considered. First, the sample size used in both the profiling and validation parts of this study was small, which restricted statistical power and may underestimate observed differences. Additionally, studying the effects of hyperglycemia on cellular events using in vitro models is challenging due to the relatively short exposure of cultured cells to high glucose conditions. This does not fully capture the complex and dynamic nature of long-term hyperglycemia-mediated changes in macrophage function.

In summary, the findings of this angiogenesis-focused study support the concept that the angiogenic status of polarized macrophages is not dichotomous along the M1–M2 axis but rather forms a continuum. Macrophages exhibit extreme functional plasticity upon exposure to different microenvironmental cues, including metabolic disorders and tumor conditions, which drive shifts in the expression of pro- and anti-angiogenic factors. The resulting endothelial response then likely depends on the balance of these mediators and the specific sensitivity of the endothelial subtype. The underlying mechanisms governing this process are highly complex and necessitate further investigation at multiple levels, including in vivo approaches at the population and single-cell level on gene expression, epigenetics, and functionality. Obtaining more comprehensive information in these areas will shed more light on macrophage behavior and facilitate advancements in our understanding of their roles in vascular complications, including those associated with diabetes and tumors.

## 4. Materials and Methods

### 4.1. Recompilation and Analysis of Macrophage Datasets

Our gene expression dataset, GSE86298, was derived from monocytes from four healthy human donors, isolated using CD14-labeled beads from buffy coats, which were then stimulated for 6 days with IFN-γ (M1), IL-4 (M2), or no additional cytokines (M0). These cells were cultivated in the presence of either 5 mM glucose (normoglycemia, NG) or 25 mM glucose (hyperglycemia, HG). The transcriptomes of the cells were measured using the Affymetrix Human Gene 1.0 ST Array [22,23,48], resulting in 24 arrays with four biological replicates for M0, M1, and M2 cultivated under both normoglycemic (NG) and hyperglycemic (HG) conditions. Similar protocols formed the basis of publications describing the influence of hyperglycemia on parameters of polarization by our research group [20,22,23].

The transcriptomic data analysis was conducted in a series of steps. First, all samples underwent quality control using the QC module from Simpleaffy (v2.26.1) [49], which utilized various parameters such as virtual image reconstruction, signal comparability, and array correlation to identify low-quality samples. All arrays passed quality control assessments, exhibiting similar RNA degradation plots and intensity distributions prior to normalization, with no detected outliers in the principal component analysis (PCA) using raw data. Data preprocessing was performed using the Limma R/Bioconductor software package (v3.64.0) [50], wherein probesets were summarized, data were normalized, and log2-transformed using the robust multichip average (RMA) algorithm.

To identify differentially expressed genes (DEGs), the linear model from Limma [50] implemented in R (v4.2.2) was used. Pairwise comparisons were made between M1 vs. M0 and M2 vs. M0 samples, and the fold change (FC), *p* value, and false discovery rate (FDR) were calculated for each probe in the microarrays. A cutoff value for DEG was set at FDR < 0.05 with a fold change ≥ |1.5|.

### 4.2. Collection of Pro- and Anti-Angiogenic Signatures

We generated a comprehensive list of genes (Supplementary Table S1) that were annotated with either pro-angiogenic or anti-angiogenic activity, utilizing both Ingenuity Pathway Analysis (IPA) (IPA, QIAGEN, version Spring 2023) [51] and the Gene Ontology Biological Processes databases (Gene Ontology Consortium, release 2023-06-01) (https://geneontology.org/).

### 4.3. Principal Component Analysis of Pro- and Anti-Angiogenic Genes in M0, M1, and M2 Macrophages

Principal component analysis (PCA) was computed on the angiogenesis-related genes defined in the IPA and GO databases—used in a complementary manner to ensure comprehensive coverage—to identify whether genes annotating anti- and pro-angiogenic signatures exhibited distinct patterns of expression among the M0, M1, and M2 samples. The following steps were undertaken for each signature (anti-angiogenic and pro-angiogenic): first, the expression levels of the various genes within each signature were selected for all the samples; second, PCA was computed; third, genes that contributed significantly to the separation of M0, M1, and M2 samples in the PCA plot were identified; and finally, the DEGs were extracted from the list of genes obtained in the previous step.

To identify the most influential genes in the clustering of the M1, M2, and M0 samples, we used the pcaGoPromoter package implemented in R (v1.0.0) [52]. We calculated the loads (genes) for each principal component, allowing for the ranking of the genes based on their importance in the distribution of the samples across the principal components.

### 4.4. PBMC Isolation

Peripheral blood mononuclear cells (PBMCs) were isolated from normal blood donor buffy coats using standard two-step Ficoll-Paque gradient centrifugation [53]. Cell pellets were resuspended in RPMI freezing medium (containing 40% fetal bovine serum (FBS, Gibco, Waltham, MA, USA) + 10% dimethyl sulfoxide (DMSO, Sigma-Aldrich, St. Louis, MO, USA)) and stored in liquid nitrogen until further use.

### 4.5. Human Monocyte Isolation

PBMCs were thawed, counted (Countess II; Thermo Fisher, Waltham, MA, USA), and labeled with MACS colloidal superparamagnetic microbeads (# 130-050-201, Miltenyi Biotec, Leiden, the Netherlands) conjugated with monoclonal anti-human CD14 antibodies for positive selection of CD14^+^ monocytes according to the manufacturer’s instructions. The purity of CD14^+^ cells (> 95% pure) was confirmed using flow cytometry.

### 4.6. Macrophage Polarization

Monocytes were cultured in RPMI-1640 medium (Gibco) supplemented with 10% FBS (Gibco). From day zero, the cells were stimulated with either M-CSF (100 ng/mL, # 130-093-491, Miltenyi Biotec), M-CSF + IL-4 (40 ng/mL, # 130-093-917, Miltenyi Biotec), M-CSF + IL-6 (50 ng/mL, # 206-IL-050/CF, R&D Systems, Minneapolis, MN, USA), or M-CSF + IFN-γ (40 ng/mL, # 285-IF-100/CF, Bio-Techne, Minneapolis, MN, USA) in standard (11 mM) or high (25 mM) glucose concentrations. After 4 days of culture, the morphology was examined at 200× magnification using an Axiovert 100 light microscope (Zeiss, Oberkochen, Germany). Next, the culture supernatant was discarded, and adhered cells were harvested for flow cytometric analysis using 30 min incubation with cold PBS (calcium- and magnesium-free) + 1 mM EDTA or prepared for mRNA isolation.

### 4.7. Flow Cytometry

Harvested cells were washed with staining buffer (MACSima^TM^ running buffer, Miltenyi Biotec, Bergisch Gladbach, Germany) and exposed to labeled anti-human monoclonal antibodies at 4 °C in a dark environment according to the manufacturer’s instructions. The following antibodies were used: CD14-PE-Cy7 (61D3, eBioscience^TM^, San Diego, CA, USA), CD16-APC-Cy7 (3G8 (RUO), BD Pharmingen^TM^, Franklin Lakes, NJ, USA), CD163-PE (GHI/61, BD Pharmingen^TM^), CD64-APC (10.1 (RUO), BD Pharmingen^TM^), anti-HLA-DR (MHC class II)-FITC (G46-6, BD Pharmingen^TM^), and CD206 (MRC1)-FITC (15-2; BioLegend, Amsterdam, the Netherlands). After 20 min of incubation, the cells were washed and resuspended in 200 µL of staining buffer. Surface marker expression was measured using a FACS Canto^TM^ II cell analyzer (BD Biosciences, Piscataway, NJ, USA). Viable cells were gated, and data were analyzed using FlowJo software (version 10.8.1, Tree Star, Ashland, OR, USA). At least 10,000 events were acquired per sample.

### 4.8. Real-Time Quantitative Polymerase Chain Reaction Analysis

After 4 days of macrophage differentiation, mRNA was isolated and converted into cDNA using a commercially available kit (GenElute Mammalian Total RNA Miniprep Kit; Sigma-Aldrich, USA). Transcript levels were determined by real-time quantitative polymerase chain reaction (QuantStudio 5 (version 1.3.1); Thermo Fisher Scientific, Waltham, MA, USA), normalized to the control gene Abelson (*ABL*), and expressed as relative mRNA expression using the ΔCT method [54]. Primer-probe combinations used are listed in Supplementary Table S2.

### 4.9. Statistics

Data obtained from flow cytometry and qPCR were analyzed using GraphPad Prism (version 5.04). To determine significant differences between different experimental conditions, Student’s T-test, the nonparametric Mann–Whitney U T-test, or one-way ANOVA followed by a post-hoc Tukey multiple comparison test were used, and a *p* value < 0.05 was considered statistically significant. All data are presented as means ± standard error of the mean (SEM).

## Figures and Tables

**Table 1:** Number of DEGs in M1 and M2 as compared to M0.

	DEG ^b^	Up ^c^	Down ^c^	Common ^d^
M1-NG vs. M0-NG ^a^	1630	898	732	
M1-HG vs. M0-NG	1753	939	814	1190
M2-NG vs. M0-NG	1423	663	760	
M2-HG vs. M0-NG	1421	695	726	1188

^a^ The M0-NG, M1-NG, and M2-NG cells were cultivated using normal concentrations of glucose, whereas M1-HG and M2-HG were cultivated with high glucose concentrations. ^b^ The DEG column indicates the total number of differentially expressed genes observed in the comparison. ^c^ Up and Down columns show the number of up-regulated and down-regulated genes. ^d^ The first number in the Common column represents the number of DEGs that are common to M1-NG and M1-HG in comparison to M0-NG, and the second number represents a similar comparison, but for M2.

**Table 2:** Pathway analysis of DEG comparing M1 vs. M0. (**A**) Regulated pathways in the list of DEGs identified in M1-NG vs. M0-NG. (**B**) Regulated pathways in the list of DEGs identified in M1-HG vs. M0-NG. The top 10 regulated pathways and the statistically significant angiogenesis pathways are shown.

**(A)**			
**Order ^a^**	**M1-NG vs. M0-NG**	**Count ^b^**	**Benjamini ^c^**
1	GO:0006954~inflammatory response	80	1.69 × 10^−13^
2	GO:0007165~signal transduction	133	0.0019
3	GO:0050728~negative regulation of inflammatory response	21	0.0016
4	GO:0051056~regulation of small GTPase mediated signal transduction	28	0.0024
5	GO:0006915~apoptotic process	75	0.0020
6	GO:0006955~immune response	60	0.0020
7	GO:0006935~chemotaxis	26	0.0023
8	GO:0001525~angiogenesis	38	0.0024
9	GO:0000082~G1/S transition of mitotic cell cycle	23	0.0028
10	GO:0002250~adaptive immune response	28	0.0065
**(B)**			
**Order ^a^**	**M1-HG vs. M0-NG**	**Count ^b^**	**Benjamini ^c^**
1	GO:0006954~inflammatory response	72	1.68 × 10^−9^
2	GO:0001525~angiogenesis	41	4.05 × 10^−4^
3	GO:0006955~immune response	61	0.0014
4	GO:0032496~response to lipopolysaccharide	31	0.0045
5	GO:0006334~nucleosome assembly	25	0.0058
6	GO:0006935~chemotaxis	25	0.0075
7	GO:0050728~negative regulation of inflammatory response	19	0.0102
8	GO:0007067~mitotic nuclear division	39	0.0099
9	GO:0050729~positive regulation of inflammatory response	18	0.0100
10	GO:0006915~apoptotic process	71	0.0094

^a^ The position of the pathway from a smaller *p* value to a larger *p* value. ^b^ The number of DEGs annotated in the respective pathway. ^c^ The corrected *p* value for multiple comparisons.

**Table 3:** Pathway analysis of DEGs comparing M2 vs. M0. (**A**) Regulated pathways in the list of DEGs identified in M2-NG vs. M0-NG. (**B**) Regulated pathways in the list of DEGs identified in M2-HG vs. M0-NG. The top 10 regulated pathways and the statistically significant angiogenesis pathways are shown.

**(A)**			
**Order ^a^**	**M2-NG vs. M0-NG**	**Count ^b^**	**Benjamini ^c^**
1	GO:0006954~inflammatory response	71	1.87 × 10^−6^
2	GO:0051607~defense response to virus	40	7.20 × 10^−6^
3	GO:0045087~innate immune response	72	6.22 × 10^−5^
4	GO:0060337~type I interferon signaling pathway	21	2.06 × 10^−4^
5	GO:0060333~interferon-gamma-mediated signaling pathway	21	0.0011
6	GO:0071222~cellular response to lipopolysaccharide	27	0.0017
7	GO:0006955~immune response	65	0.0023
8	GO:0006919~activation of cysteine-type endopeptidase activity involved in apoptotic process	22	0.0024
9	GO:0002576~platelet degranulation	25	0.0022
10	GO:0002250~adaptive immune response	31	0.0028
21	GO:0001525~angiogenesis	37	0.0316
**(B)**			
**Order ^a^**	**M2-HG vs. M0-NG**	**Count ^b^**	**Benjamini ^c^**
1	GO:0051607~defense response to virus	48	2.96 × 10^−9^
2	GO:0006954~inflammatory response	78	2.55 × 10^−8^
3	GO:0045087~innate immune response	79	3.50 × 10^−6^
4	GO:0006955~immune response	74	5.98 × 10^−5^
5	GO:0006952~defense response	22	2.48 × 10^−4^
6	GO:0060337~type I interferon signaling pathway	21	4.18 × 10^−4^
7	GO:0060333~interferon-gamma-mediated signaling pathway	22	5.23 × 10^−4^
8	GO:0009615~response to virus	27	0.0027
9	GO:0045071~negative regulation of viral genome replication	15	0.0032
10	GO:0002250~adaptive immune response	32	0.0040
24	GO:0001525~angiogenesis	38	0.0475

^a^ The position of the pathway from a smaller *p* value to a larger *p* value. ^b^ The number of DEGs annotated in the respective pathway. ^c^ The corrected *p* value for multiple comparisons.

**Table 4:** The number of differentially expressed pro- and anti-angiogenic genes in macrophage polarization under normoglycemic conditions.

	M1(IFN-γ) vs. M0		M2(IL-4) vs. M0	
	up	down	up	down
Pro-angiogenic genes	22 (7%) ^a^	39 (13%)	41 (14%)	30 (10%)
Anti-angiogenic genes	8 (7%)	9 (8%)	12 (11%)	21 (18%)

^a^ The number (% of total) of significant DEGs annotated as pro- (n = 301) or anti-angiogenic (n = 114) in the angiogenic pathway in the Gene Ontology and Ingenuity Pathway Analysis databases.

**Table 5:** Number of differentially expressed pro- and anti-angiogenic genes in macrophage polarization under hyperglycemic compared to normoglycemic conditions.

	M1(IFN-γ)-HG vs. M1(IFN-γ)-NG		M2(IL-4)-HG vs. M2(IL-4)-NG	
	up	down	up	down
Pro-angiogenic genes	48 (16%) ^a^	28 (9%)	50 (17%)	65 (22%)
Anti-angiogenic genes	15 (13%)	18 (16%)	22 (19%)	15 (13%)

^a^ The number (% of total) of significant DEGs annotated as pro- (n = 301) or anti-angiogenic (n = 114) in the combined angiogenic pathway in the Gene Ontology and Ingenuity Pathway Analysis databases.

## Data Availability

The original data presented in this study are openly available at https://www.ncbi.nlm.nih.gov/geo/query/acc.cgi?acc=GSE86298, accessed on 12 July 2017.

## Supplementary Materials

The supporting information can be downloaded at: https://www.mdpi.com/article/10.3390/ijms26104846/s1.
