# Supplementary material for: Polarized Macrophages Show Diverse Pro-Angiogenic Characteristics Under Normo- and Hyperglycemic Conditions
Source: Int J Mol Sci. 2025 May 19;26(10):4846. doi: 10.3390/ijms26104846 (PMC12111939; doi:10.3390/ijms26104846)
Supplement: Supplementary file 1 [file ijms-26-04846-s001.zip › ijms-3522750-supplementary.pdf]

(a)

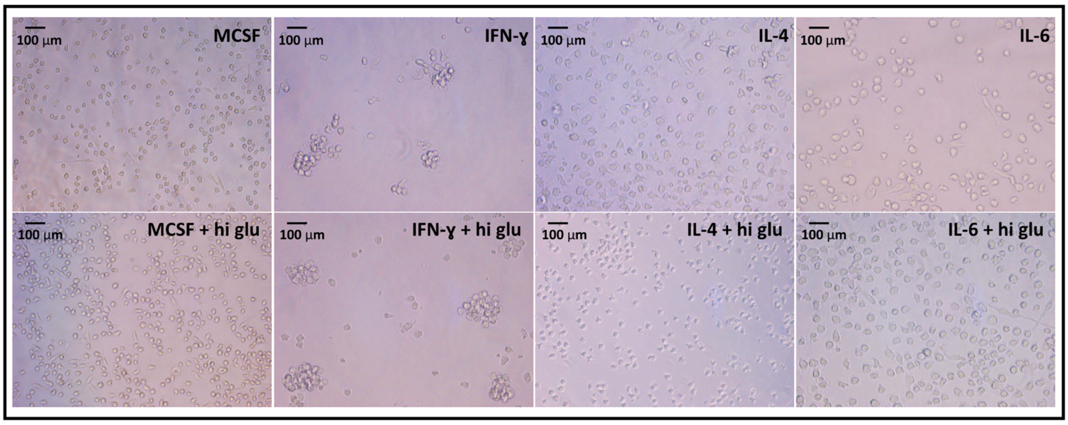

(b)

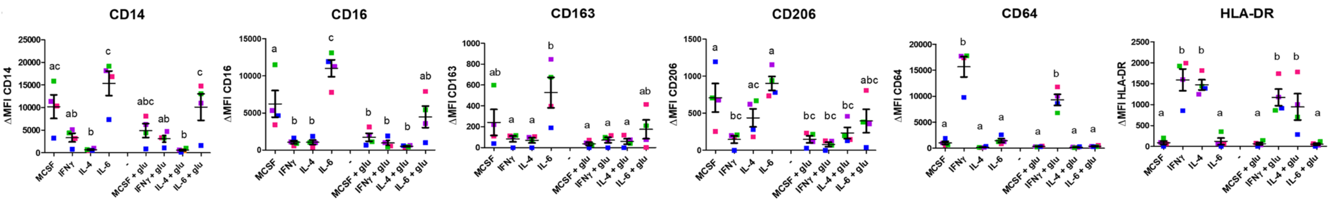

Supplementary Figure S1. Different immunophenotypes of macrophage subsets in standard- and hyperglycemic conditions. Monocytes were isolated from healthy controls and were stimulated to differentiate into M0, M1 and M2 macrophages under standard- and hyperglycemic conditions. **(a)** Bright field microscopy of different macrophage subsets, 32× magnification. **(b)** Expression of CD14, CD16, CD64, HLA-DR, CD163, and CD206 on differently polarized macrophages. Marker expression was measured by flow cytometry and expressed as median fluorescence intensity (MFI). Values were corrected for auto-fluorescence with the MFI of the backbone control (labeled with CD14 and CD16 only), or unlabeled cells (for CD14 and CD16). Error bars represent means ± standard error of the mean (SEM). Different letters indicate significant differences. Significance was calculated using one-way ANOVA and Tukey post-hoc test correction for multiple comparisons ($P < 0.05$).

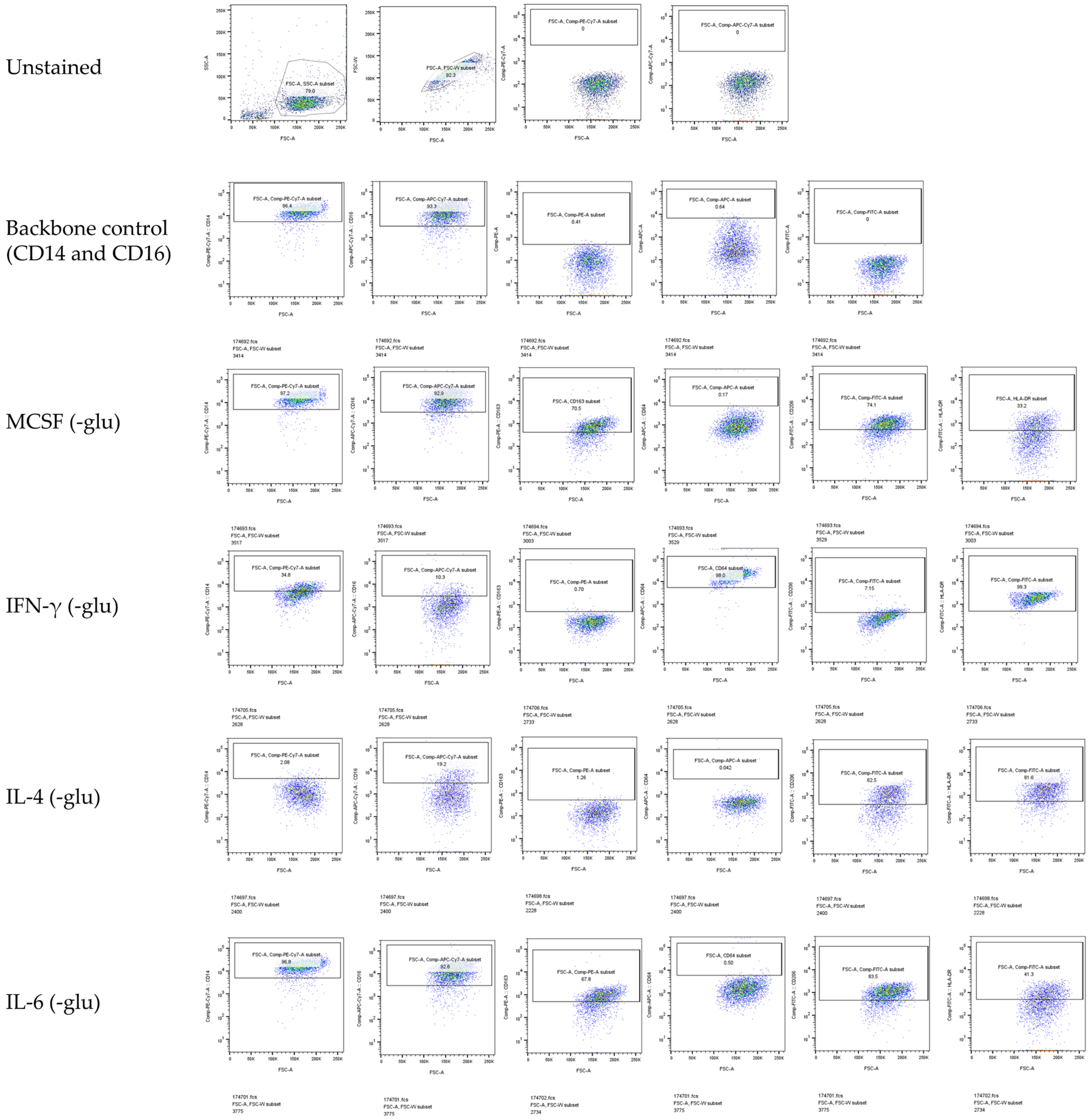

Supplementary Figure S2. Representative gating strategy for flow cytometric analysis of polarized macrophages in standard glycemic condition. Forward scatter (FSC) x side scatter (SSC) gating was used to obtain mononuclear cells based on size and granularity and exclude debris. Cell aggregate was excluded, and the representative marker expressions were plotted against the FSC. Identical gates were used for auto-fluorescence compensation (-glu = low glucose concentration).

Supplementary Table S1. A comprehensive list of genes that were annotated with either pro-angiogenic or anti-angiogenic activity, utilizing both Ingenuity Pathway Analysis (IPA) [51] and the Gene Ontology Biological Processes databases.

**Pro-angiogenic genes**							**Anti-angiogenic genes**		
ADAM9	CCL5	EPHB2	HMGB2	LDHA	PDGFB	SER-PIND1	ADA	IKBKB	SBDS
ADM2	CCL7	EPHB3	HMOX1	LEPR	PDGFC	SER-PINH1	ADAMTS12	IL12A	SEMA3E
AGGF1	CCR3	EPHB4	HP	LGALS3	PDGFD	SFRP1	ADAMTS2	IL13RA2	SEMA3F
AKR1B1	CD34	EPO	HSF1	LGALS8	PIGF	SFRP2	ADAMTS8	IL17F	SER-PINB5
AKT3	CD40LG	ERAP1	HSP90AA1	LOX	PIK3C2B	SHH	ADGRB1	IL1RN	SER-PINC1
AMELX	CD63	ERBB2	HSPA5	LRP8	PIK3R6	SMOC2	AIMP1	IL2	SHROOM2
ANGPTL2	CDH1	EREG	HSPB1	LRPAP1	PKM	SRPX2	ANGPTL1	IL24	SLIT3
ANGPTL3	CDH13	ETS1	HSPB6	MAML1	PLAU	ST6GAL1	APC	IL27	SLURP1
ANPEP	CDH2	F12	HSPG2	MAPK1	PLAUR	STAT3	APOE	JUP	SMPD1
ANXA2	CDH5	F2	HTRA1	MAPK14	PLCG1	STC1	APOH	KCNJ2	SOD1
ANXA3	CEA-CAM1	F2R	HYAL1	MAPK7	PLXDC1	STX6	APP	KLK3	SPINK5
AOC3	CELA1	F3	ICAM1	MAP-KAPK2	PLXNB1	TAB2	ATP6V0A2	KRIT1	SPINT1
AP1S2	CHI3L1	FABP4	IGF1R	MCAM	PLXNB3	TAC1	BMPER	LAMA5	SST
APLN	CMA1	FAS	IGF2R	MCF2L	PPIA	TFEB	BPI	LIF	STARD13
APOA1	COMP	FERMT2	IHH	MDK	PRKCE	TGFA	CALR	LRP1	STAT1
AQP1	CREBBP	FERMT3	IL17A	MERTK	PROC	TGFBR3	CASP1	MB	SULF1
AREG	CRYAB	FES	IL17B	MET	PROCR	TGM2	CAT	MED1	TF
ARG1	CSF1	FGF1	IL17C	MFGE8	PROK1	THBS4	CD82	MMP12	TFPI
ATF2	CSF3	FGF10	IL18BP	MGAT5	PROK2	TJP1	CDKN1A	MYH10	TGFBI

ATF3	CSH1	FGF13	IL19	MGP	PTGIS	TLR2	CHGA	NAB2	THBD
ATM	CSH2	FGF3	IL1A	MIB1	PTGS2	TNC	CNMD	NEU1	THBS2
AXL	CSNK1A1	FGF4	IL20	MMP1	PTH	TNFSF11	COL18A1	NGFR	TIMP2
B4GALT1	CSPG4	FGF5	IL25	MMP13	PTK2B	TRPC4	COL4A1	NPPA	TIMP3
BDNF	CTGF	FGF6	IL3	MMP14	PTPRB	TRPC6	COL4A2	NPPB	TNFRSF25
BECN1	CTSB	FGF7	IL4R	MMP3	PTPRZ1	UTS2	COL4A3	NPPC	TNFSF15
BMP2	CTSH	FGF8	IL6	MTOR	RAC1	VAV3	CST3	NRG2	TNNI3
BMP4	CTSS	FGF9	IL6R	MUC4	RAMP1	VCAM1	CXCL10	OPTC	VLDLR
BMP6	CX3CL1	FGFR1	INSR	MYDGF	RB1	VEGFA	CXCL14	PDGFA	XIAP
BMPR2	CXCL1	FGR	IRS1	MYH9	RDX	VEGFB	CXCL9	PF4	
BMX	CXCL12	FLNA	ITGA4	MYOF	RLN2	VEGFC	DCN	PF4V1	
BRAF	CXCL2	FLNB	ITGA9	NCKIPSD	ROBO1	VEGFD	DKK1	PGK1	
BRCA1	CXCL5	FOLH1	ITGAM	NDNF	RPSA	VIM	DMP1	PLXND1	
BTC	CXCL6	FURIN	ITGAV	NELL1	S100A12	VIP	FBLN2	PRKCD	
C1QA	CXCL8	G6PD	ITGB4	NGF	S100A4	WNK1	FSHR	PRKDC	
C5	CYP11B2	GAST	JAG1	NODAL	S100A8	WNT2	GHRL	PRLH	
C6	CYSLTR2	GH2	JAM3	NOS1	S100A9	WNT5A	GRK6	PROS1	
CAMP	ECM1	GPC1	KIT	NOTCH1	SAA1	WNT7B	GSN	PTPN6	
CCBE1	EDIL3	GREM1	KITLG	NOX4	SASH1	YARS	GTF2I	PTPRM	
CCL1	EGFL6	GRN	KMT2A	NPY	SELE	YWHAZ	HABP2	PTX3	
CCL11	EGFL7	HAS3	L1CAM	NRG1	SELL	YY1	HSPD1	RBL2	
CCL24	EGFR	HBEGF	LAMA2	ORM1	SEMA3D	ZC3H12A	IFNA2	RECK	
CCL26	EGR1	HCK	LAMC1	OXT	SEMA4D	ZC3H13	IGFBP4	ROCK1	
CCL28	ENPP2	HMGB1	LCN2	PATJ	SEMA5A	ZG16B	IGHG1	SARS	

Supplementary Table S2. Primer-probe combinations used for qPCR.

Gene	Forward primer, 5′-3′	Reverse primer, 3′-5′	Probe, 5′ FAM-3′ TAMRA/MGB-NFQ
**ABL**	TGGAGATAACATCTAA-GCATAACTAAAGGT	GATGTAGTTGCTT GGGACCCA	CCATTTTTGGTTTGGG CTTCACACCATT
**S100A8**		TaqMan Gene Expression Assays (4331182 Hs00374264_g1) Life Technologies	
**CXCL8 (IL-8)**		TaqMan Gene Expression Assays (4331182 Hs00174103_m1) Life Technologies	
**FABP4**		TaqMan Gene Expression Assays (4331182 Hs01086177_m1) Life Technologies	
**HTRA1**		TaqMan Gene Expression Assays (4331182 Hs01016151_m1) Life Technologies	
**MERTK**		TaqMan Gene Expression Assays (4331182 Hs01031979_m1) Life Technologies	
**VEGF-A**		TaqMan Gene Expression Assays (4331182 Hs00900055_m1) Life Technologies	
**JAG1**		TaqMan Gene Expression Assays (4331182 Hs01070032_m1) Life Technologies	
**CDH1**		TaqMan Gene Expression Assays (4331182 Hs01023895_m1) Life Technologies	
**CCL5**		TaqMan Gene Expression Assays (4331182 Hs00982282_m1) Life Technologies	
**FAS**		TaqMan Gene Expression Assays (4331182 Hs00236330_m1) Life Technologies	
**CCL28**		TaqMan Gene Expression Assays (4331182 Hs00219797_m1) Life Technologies	
**CXCL9**		TaqMan Gene Expression Assays (4331182 Hs00171065_m1) Life Technologies	
**STAT1**		TaqMan Gene Expression Assays (4331182 Hs01013996_m1) Life Technologies	
**PTX3**		TaqMan Gene Expression Assays (4331182 Hs00173615_m1) Life Technologies	
**TIMP3**		TaqMan Gene Expression Assays (4331182 Hs00165949_m1) Life Technologies	

**TGFB1**	TaqMan Gene Expression Assays (4331182 Hs00998133_m1) Life Technologies
**CDKN1A**	TaqMan Gene Expression Assays (4331182 Hs00355782_m1) Life Technologies
**THBD**	TaqMan Gene Expression Assays (4331182 Hs00264920_s1) Life Technologies

Supplementary Table S3. Selected genes for validation experiments. (A) Pro-angiogenic genes (B) Anti-angiogenic genes.

A.

**Pro-angiogenic**	**PC1 positive**	**PC1 negative**	**PC2 positive**	**PC2 negative**	**Role in angiogenesis**
FABP4	✓	-	-	-	Promotes endothelial cell proliferation and tube formation.
CDH1	✓		-	-	Facilitates endothelial barrier integrity; indirectly supports angiogenesis.
CCL28	✓				Recruits endothelial and immune cells to support neovascularization.
S100A8		✓			Stimulates endothelial cell migration and VEGF production.
CXCL8		✓			Strongly pro-angiogenic chemokine; induces endothelial proliferation and migration.
CCL5		✓			Enhances endothelial cell chemotaxis and angiogenesis via CCR5.
JAG1			✓		Activates Notch signaling in endothelial cells to promote vessel sprouting.
FAS			✓		Involved in vascular remodeling; paradoxical role in angiogenesis depending on context.
HTRA1				✓	Regulates extracellular matrix remodeling during angiogenesis.
MERTK				✓	Promotes phagocytosis and tissue repair; supports angiogenesis in inflammation.

B.

**Anti-angiogenic**	**PC1 positive**	**PC1 negative**	**PC2 positive**	**PC2 negative**	**Role in angiogenesis**
PTX3	✓				Inhibits FGF2 activity and endothelial proliferation.

TIMP3	✓				Inhibits MMPs and VEGF signaling, suppressing angiogenesis.
THBD	✓				Limits inflammation-induced angiogenesis via anticoagulant properties.
CXCL9		✓			Suppresses endothelial cell proliferation and migration.
BPI		✓			Binds LPS and inhibits inflammatory angiogenesis.
STAT1		✓			Inhibits angiogenesis via suppression of VEGF signaling.
CDKN1A			✓		Cell cycle regulator that limits endothelial proliferation and vessel formation.
PDGFA			✓		Dysregulated PDGFA can impair vessel maturation and contribute to non-productive angiogenesis in pathological settings.
ADA				✓	Linked to reduced angiogenesis by modulating adenosine signaling.
TGFBI				✓	Promotes ECM stabilization, which can limit angiogenic.
